# Heuristic Modeling of Carcinogenesis for the Population with Dichotomous Susceptibility to Cancer: A Pancreatic Cancer Example

**DOI:** 10.1371/journal.pone.0100087

**Published:** 2014-06-16

**Authors:** Tengiz Mdzinarishvili, Simon Sherman

**Affiliations:** Eppley Institute for Research in Cancer, University of Nebraska Medical Center, Omaha, Nebraska, United States of America; University of Torino, Italy

## Abstract

At present, carcinogenic models imply that all individuals in a population are susceptible to cancer. These models either ignore a fall of the cancer incidence rate at old ages, or use some poorly identifiable parameters for its accounting. In this work, a new heuristic model is proposed. The model assumes that, in a population, only a small fraction (pool) of individuals is susceptible to cancer and decomposes the problem of the carcinogenic modeling on two sequentially solvable problems: (i) determination of the age-specific hazard rate in individuals susceptible to cancer (individual hazard rate) from the observed hazard rate in the population (population hazard rate); and (ii) modelling of the individual hazard rate by a chosen “up” of the theoretical hazard function describing cancer occurrence in individuals in time (age). The model considers carcinogenesis as a failure of individuals susceptible to cancer to resist cancer occurrence in aging and uses, as the theoretical hazard function, the three-parameter Weibull hazard function, often utilized in a failure analysis. The parameters of this function, providing the best fit of the modeled and observed individual hazard rates (determined from the population hazard rates), are the outcomes of the modeling. The model was applied to the pancreatic cancer data. It was shown that, in the populations stratified by gender, race and the geographic area of living, the modeled and observed population hazard rates of pancreatic cancer occurrence have similar turnovers at old ages. The sizes of the pools of individuals susceptible to this cancer: (i) depend on gender, race and the geographic area of living; (ii) proportionally influence the corresponding population hazard rates; and (iii) do not influence the individual hazard rates. The model should be further tested using data on other types of cancer and for the populations stratified by different categorical variables.

## Introduction

The purpose of carcinogenic modeling is to increase our understanding of the processes leading to cancer development in time (carcinogenesis). The modeling allows one to raise questions and yield predictions, which could be validated (or refuted) in new biomedical experiments [1]. A better understanding of carcinogenesis can help researchers generate and test new hypotheses as well as develop improved strategies for cancer prevention. Throughout the nearly 60 years of carcinogenic modeling history, a large body of different models has been proposed (see, for instance, [2]–[14] and references therein).

Mathematically, a problem of the carcinogenic modeling is stated as the best fitting of the modeled cancer hazard rate with the observed rate. To solve this problem, the existing models use the “bottom-up” computing framework, which requires knowledge of a mechanism of cancer occurrence in individuals susceptible to cancer (individual level) in a time (age) scale. Since such a mechanism is not well-known yet, researchers initially conjecture a plausible mechanism and describe it mathematically by the corresponding formulas. Using these formulas, researchers calculate the cancer hazard rate in a population (population hazard rate) and, by the calculated rate, fit the cancer hazard rate observed for the population. Parameters of the used formulas that provide the best fit for the observed population hazard rate are taken as the final result of modeling. When the fit is not good enough, or when the obtained values of the parameters do not agree with the current biological knowledge, the researchers “adjust” the initially conjectured mechanism of carcinogenesis (mathematical presentation by the corresponding formulas) and repeat the modeling.

The modern carcinogenic models imply that, for all individuals in the population, getting cancer is a certain event, i.e. they assume “cancer is inevitable for those who live long enough”. Some of these models (such as [2]–[6]) assume that all individuals in the population are equally susceptible to cancer, while others ([7]–[12]) assume that individuals have different susceptibility to cancer (due to unobserved random factors) and introduce a non-negative random variable (a frailty). The use of frailty allows researchers to get a better fitting, but requires additional parameters, characterizing the frailty distribution. These parameters, however, do not always have clear biological meaning. To improve fitting, researchers also implement more and more biological details in the mechanisms of cancer occurrence in individuals [5]–[6]. However, the use of advanced biological mechanisms makes the modeling a very complicated computational problem with poorly identifiable parameters [12]. In other words, researchers attempt to “replace the biological system we are trying to understand by a huge computational model that we have no chance of ever understanding!” [1].

Current carcinogenic models poorly utilize the fact that, for many individuals in the population, getting cancer is not a certain event: in the population, a big fraction of individuals are resistant to cancer and did not get cancer in their lifetime, while only a small fraction (pool) of individuals from the population are susceptible to cancer and individuals from this pool eventually will get cancer. It should be noted that even when the population is heavily exposed to known chemical carcinogenic agents, less than 20% of the population can develop a particular type of cancer [13]. For the majority of cancer types, the size of the pool of individuals susceptible to cancer does not exceed several percent [10].

The main goal of this work is to develop a novel approach for carcinogenic modeling that will fully use the observation that cancer is a rare disease. Some components of the proposed approach were published in [15]–[17]. The approach uses a hypothesis of the dichotomous susceptibility to cancer in the population. This hypothesis was initially suggested and rejected in [14]. Therefore, in the present work, formulas and data presented in [14] were audited to check a validity of the hypothesis. The proposed approach was applied for modeling of pancreatic cancer occurrence using data, collected in the Surveillance, Epidemiology, and End Results (SEER) databases [18].

## Materials and Methods

### Terminology, Notations and a General Statement of the Problem

Usually, parameters of carcinogenic models are determined by the frequency of cancer occurrence in populations. For this purpose, the age-specific incidence rate (crude rate), characterized by a number of cases with a distinct type of cancer within the age-specific population (the population of individuals equally distributed in specified age intervals) are often used. The age-specific incidence rates are determined as a ratio of the observed number of cancer cases, divided by the total person-years at risk, in the population of individuals within distinct age intervals (often taken as the sequential, five-year long age intervals) of the human lifespan [19]. Since cancer is a rare disease, the age-specific incidence rates are collected during a long time period for individuals from different birth cohorts. From the observed age-specific incidence rates of cancer, the estimates of the age-specific hazard rates in the specified age intervals can be calculated by using the age-period-cohort (APC) analysis [3]–[5], [16], [20]. The age-specific cancer hazard rate obtained in such a way is referred to as the population hazard rate [17]. Analogously, the age-specific cancer hazard rate determined in the considered age intervals of human life for an individual susceptible to cancer are referred to as the individual hazard rate [17].

In this work, for the convenience of mathematical presentation, the concepts of the population and individual (theoretical) hazard functions are used along with the population and individual hazard rates. For the population and individual hazard functions, the age 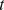 is a continuous variable, while for the population and individual hazard rates, the age interval 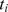 is a discrete variable presenting the corresponding 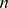 successive age intervals with indexes 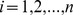.

### Mathematical Relationship between the Population and Individual Hazard Functions

Let us denote by 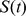 a conditional survival function that an individual “survives” from getting a particular type of cancer at the age 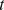, given this individual belongs to the pool of individuals susceptible to cancer. For individuals not susceptible to cancer, the conditional survival function will be equal to 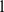 at any age. Let us also denote by 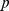 the probability (portion of the pool within the population) that a randomly chosen individual belongs to the pool of individuals susceptible to cancer. Then, 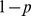 will be the probability that this individual belongs to the pool of individuals not susceptible to cancer. According to [21], the unconditional survival function (or population survival function) 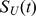 that an individual, randomly chosen from the population, survives from cancer at the age 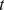 will be:
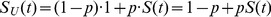
(1)and



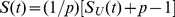
(2)In survival analysis, the hazard function (theoretical hazard function, *thf*) 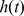, the probability density function 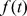, and the survival function 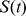 are related by the following equations [21]:
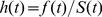
(3)

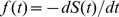
(4)and
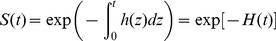
(5)where 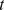 denotes a specified value of the survival time random variable and
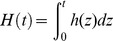
(6)is the cumulative individual hazard function.

From formulas (1)–(6) it follows that the unconditional (population) hazard function 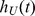 of an individual, randomly chosen from the whole population, gets cancer at the age 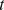 is:
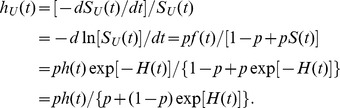
(7)


From these formulas, it follows that, for an individual randomly chosen from the pool of individuals susceptible to cancer, the hazard function, 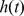, of getting cancer at the age 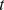 is:
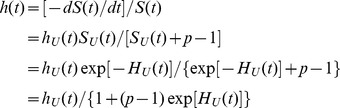
(8)where:
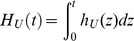
(9)is the cumulative population hazard function.

Note, from the formula (7) it follows that if 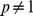 and 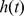 tends to infinity, when 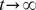 then 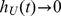. In other words, when the individual hazard function increases at old ages, the population hazard function falls.

From (7) and (1) it follows:
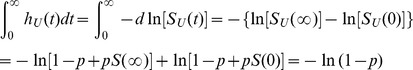
(10)Note, for the aforementioned designations: 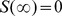 and 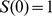.

When 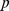
(the size of the pool of individuals susceptible to cancer) is small, then, with a first-order approximation, the overall cumulative hazard 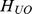 can be presented as:
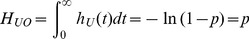
(11)


Also note that, for small 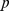 (i.e. when 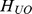 is small), formulas (7) and (8) can be presented with a first-order approximation as:
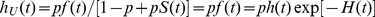
(12)

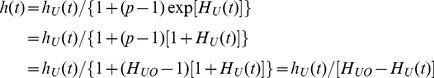
(13)


For cancer, the overall cumulative population hazard 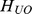 is small (for example, for pancreatic cancer, 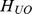
<0.01). Therefore, for cancer, the formula (13) can be used for assessing 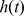 from 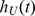. An empirical estimate 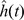 (here and below the sign “∧” designates an empirical estimate), can be presented as:
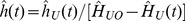
(14)


Using standard rules of error propagation [22], for the standard errors (*SE*) of the estimates of the *thf*, 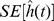, we obtained:
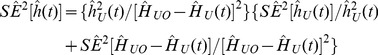
(15)


Note, in the present work, formulas (14)–(15) were derived in a simpler way compared to that made in [17]. In this connection, it needs to be pointed out that in the right sides of the formulas (20) and (40) presented in [17] there are typos (to be correct, the right sides of these formulas need to be inverted), which do not influence the other formulas and results presented in [17].

### Computing Framework for Carcinogenic Modeling in the Population with Dichotomous Susceptibility to Cancer

In the present work, for individuals susceptible to cancer, carcinogenic modeling is performed by a “top-down” computing framework that includes the following four steps:

1. To determine the estimates of a size of the fraction of individuals susceptible to cancer in the population (i.e. overall cumulative population hazard), 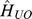, its standard error, 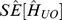, cumulative population hazard rate 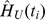 and 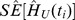) *via* 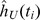 and 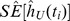.
2. To determine the estimates of the individual hazard rates, 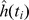, and their 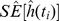 *via* 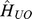, 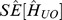, 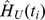 and 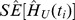.
3. To choose “up” an appropriate mathematical form of the theoretical (individual) hazard function, 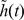.
4. To determine the values of the 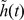 parameters that best fit the 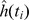 obtained in Step 2 and ascertain their consistence with the current biological knowledge.

In Step 1, the size of the fraction of individuals susceptible to cancer, 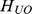, can be easily determined by formula (11). It is important to emphasize that 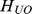 can be interpreted as a probability *p* that an individual, randomly taken from the considered population, is susceptible to cancer. The estimate 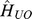 can be obtained *via* the estimates, 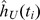.

In Step 2, the estimates of the individual hazard rates, 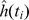, and their standard errors, 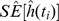, are determined using formulas (14)–(15). Note, 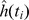
and 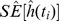 are obtained without the use of any detailed information on the carcinogenic mechanisms given up-front.

In Step 3, a plausible candidate for the theoretical hazard function 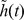 is chosen “up”. As such candidates, the functions that already have been used in popular carcinogenic models (such as, [2]–[6]) or some other functions can be taken. For instance, as a plausible candidate for 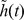, a Weibull hazard function, often used in survival analysis [21], can be utilized.

In Step 4, the parameters of the considered 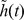 that best fit the 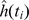
are determined by methods of linear or nonlinear regression analysis [23]. Since the fitting is performed on the individual level (for the fraction of individuals equally susceptible to cancer), no additional assumptions on cancer susceptibility are needed. The goodness of fitting can be estimated by the Akaike’s information corrected criterion (AIC). Assuming that the scatter of points around the regression line follows a Gaussian distribution, the AIC can be defined by the following formula [17]:
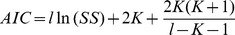
(16)where (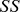) is the weighted sum of square deviations of the observed points from the obtained regression line, where 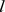 is the number of observed points, and 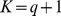 (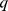 is the number of parameters used for curve fitting).

The consistence of the values of these parameters with the current biological knowledge is ascertained. For instance, the value of the parameter presenting a number of mutations needed for a normal cell to become a malignant cell should be within the interval of 2–7 because the bigger number of mutations will be hardly achievable during a human lifetime [3]–[4].

### Reviving the Rejected Hypothesis of Dichotomous Susceptibility to Cancer in the Population

The proposed “top-down” computing framework can be utilized when the hypothesis of dichotomous susceptibility to cancer in the population is correct. However, this hypothesis was considered in [14] and was ultimately rejected by its authors. Here, we argue that the reason for this rejection was erroneous, and, thus, this hypothesis should be revived.

In the frame of the multi-mutation model of carcinogenesis [2] (i.e. when 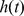 is an exponential function), the authors of [14] studied how the shapes of the curves of the cancer age-specific incidence rates depend on the sizes of the pools of individuals susceptible to cancer, 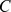
(in our designation 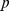), in the dichotomous, susceptible to cancer population. According to their calculations, the cancer incidence rates have turnovers with peaks at different ages depending on the sizes of the pools of individuals susceptible to cancer (see Figure 1 in [14]). Thus, that analysis suggested that peaks and falls of the cancer incidence rates of rarer cancers should appear at a younger age. However, such analysis is inconsistent with the observational data showing that the ages at which peaks and falls of the age-specific incidence rate are appearing, are independent of the rareness of the corresponding cancer types [14], [24]. Due to this inconsistency, the hypothesis of dichotomous susceptibility to cancer in the population was rejected in [14].

**Figure 1:**
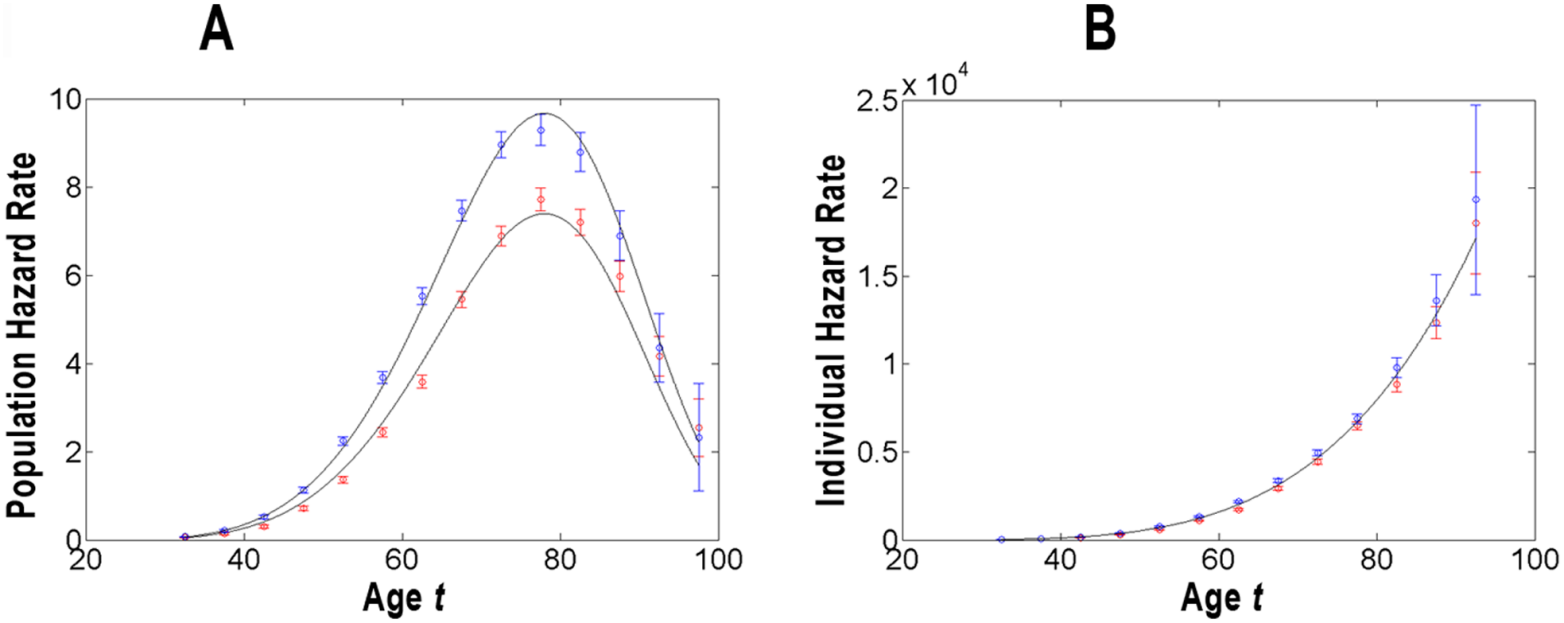
Modeling of the population (panel A) and individual (panel B) hazard rates of pancreatic cancer in men and women. For these populations, the estimates of the rates and their 95% the confidence interval (*CI*) are given in units of number of cancer cases per 100,000 person-years and presented by circles and error bars of blue (for men) and red (for women) vs. age in years. The modeled population and individual hazard functions are presented by solid lines.

Below, we demonstrate that this inconsistency is accrued due to a mistake in formula (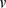) that was used in [14] for testing this hypothesis. To prove this, the aforementioned formula (7) is rewritten in the logarithmic form:
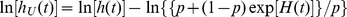
(17)


The formula (17) should be similar to formula (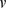) presented in [14]. However, formula (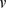) rewritten in the notations used in the present work is:
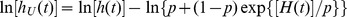
(18)


As can be easily seen, formula (17) and formula (18) are different only in the placement of brackets. In the present work, the corresponding calculations were performed by the formulas (17) and (18) (data are not shown). Interestingly, the calculations performed by formula (18) qualitatively repeat the results presented in [14]. However, calculations performed by formula (17), which are different from the calculations obtained by formula (18) suggest that the pool sizes, 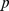
_,_ proportionally influence the population hazard function, but the ages at which peaks and falls of these functions take place are nearly the same for different 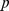. This is consistent with the observed data (see below Results). Based on this comparison, one can suggest that the hypothesis that the population has a dichotomous susceptibility to cancer was erroneously rejected in [14].

### Preparation of Pancreatic Cancer Data

In this work, to provide new observational evidence of dichotomous susceptibility to cancer in the population, the PC data collected the SEER9 databases [18] from 1975 until 2004 on the population living in nine geographical areas (Atlanta, CT, Detroit, IA, Bay area, Seattle, HI, NM, UT) was used. For the purpose of convenience, the PC data collected in nine geographic areas were divided on two datasets called “Eastern” and “Western”. PC data collected in Atlanta, CT, Detroit and IA were assigned to the Eastern dataset, while data collected in the Bay area, Seattle, HI, NM and UT were assigned to the Western dataset. Only data on patients diagnosed with the first primary, microscopically-confirmed PC were used. The use of such data in survival analysis of the PC was recommended in [25].

For extraction of data and for primary data processing, the statistical software package, SEER*Stat version 8.0.4, was used. With this software, the age-specific incidence rates collected during 30 years (1975–2004) for populations stratified by gender (men and women), race (black and white), and geographical area (Eastern and Western) were determined. Data were combined in six (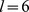), five year-long (cross-sectional) time-period intervals (197–1979; 1980–1984; 1985–1989; 1990–1994; 1995–1999; and 2000–2004). Since the number of the PC cases in individuals younger than 30 years old was too small for statistical analysis, cases only for individuals diagnosed with PC at age 30 and older were utilized.

The chosen PC cases were fractioned into 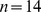 groups, corresponding to the five year-long age intervals, 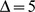 years, ranging from 30 to 99 years old. For each of these age groups (noted by 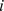 with the midpoint 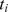 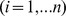) and for each of the six considered time-period intervals (noted by 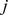 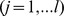) the age-specific incidence rates 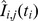, as well as their standard errors 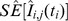 were estimated as:
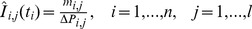
(19)

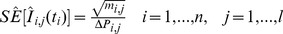
(20)In (19) and (20), 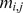 and 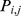 are the number of cancer cases and the size of population in the 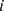-th age interval, observed during the 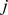-th time-period, correspondingly.

The 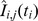 were used to estimate the population hazard rate 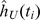. In a general case, the 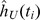 should be obtained by the use of the age-period-cohort analysis [3]–[5], [14], [15]. However, in [17] it was found that the time-period and birth-cohort effects for the age-specific incidence rates of PC data are negligible small. Therefore, in the present work, 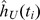 were obtained as the weighted means of the age-specific incidence rates:
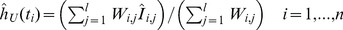
(21)where weights, 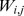 were calculated as
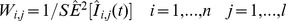
(22)and



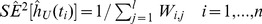
(23)The estimates of the theoretical (individual) hazard rates, 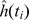, and the estimates of their standard errors, 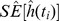, were obtained using the equations (14)–(15), which were presented by formulas:
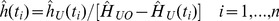
(24)

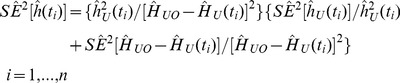
(25)


The estimates of the cumulative population hazard rate, 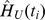, the overall cumulative population hazard, 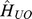, and the values of the 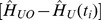 (as well as the 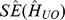 and 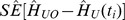) were obtained *via* the 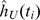 and 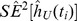, given by formulas (21) and (23), and by using the finite sums with the step 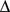 for approximation of the integrals (9) and (11), performed in a standard way [17].

### Modeling the Population and Individual Hazard Functions in the Stratified Populations

In this work, for modeling the individual hazard function, 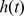, a three-parametric Weibull function is utilized [17]:
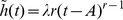
(26)where *λ* - an average number of clones developed from the mutated cells during the first year after the beginning of the effective period of the cancer exposure, *r -* a number of mutations needed to transform a normal cell into a malignant one, and *A* – a time shift (in years) that may include a period between birth and the age at the beginning of carcinogenesis, as well as an average time needed for clonal expansion of malignant cells into the clinically detectable tumor [14].

Assuming that, for the stratified populations, the individual hazard function 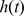 is the same as the individual hazard function for the unstratified population, one can obtain:
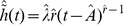
(27)


In this case, for the stratified populations, the population hazard function 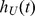 has the following form:
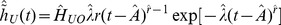
(28)


The formula (28) follows from the formulas from (12) and (27). In (28), for 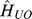 the corresponding estimates obtained for the stratified populations are used.

## Results and Discussion

The heuristic approach and the corresponding four steps computing framework, described in Materials and Methods, were used for modeling of carcinogenesis in the pancreas. The required data were extracted from the SEER 9 databases [18]. For carcinogenic modeling, seven sets of data were prepared (see Materials and Methods): one set of data for the unstratified population and six sets of data for the populations stratified by gender (male, female), race (black, white) and geographic areas of living (Eastern, Western). Since the results of the carcinogenic modeling for the unstratified population were reported in [17], mainly results for the stratified populations are presented.

For the stratified populations, the estimates of the population incidence rates, 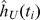, and their standard errors, 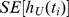, in units of number of cancer cases per 100,000 person-years obtained from SEER data, are shown in Tables 1–6. From these estimates, the sizes of the fraction of individuals susceptible to cancer (i.e. overall cumulative population hazards, 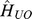) and their standard errors (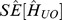), as well as the estimates of the corresponding cumulative population hazard rates, 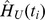, and their 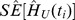) were determined as described in Step 1 of the proposed computing framework.

**Table 1:** Estimates of the population and individual hazard rates (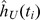 and 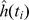, correspondingly) and their standard errors (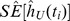 and 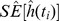) of PC occurrence in men.

Age intervals		Estimates of the hazard rates and their standard errorsa)			
Index	Middle pointb)	Population level		Individual level	
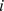	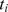	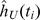	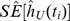	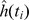	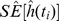
1	32.5	7.14E−02	7.26E−03	2.32E+01	2.37E+00
2	37.5	2.13E−01	1.30E−02	6.95E+01	4.25E+00
3	42.5	5.23E−01	2.15E−02	1.72E+02	7.06E+00
4	47.5	1.13E+00	3.39E−02	3.77E+02	1.13E+01
5	52.5	2.24E+00	5.09E−02	7.68E+02	1.75E+01
6	57.5	3.68E+00	7.05E−02	1.33E+03	2.55E+01
7	62.5	5.52E+00	9.40E−02	2.17E+03	3.73E+01
8	67.5	7.46E+00	1.20E−01	3.37E+03	5.53E+01
9	72.5	8.95E+00	1.48E−01	4.96E+03	8.59E+01
10	77.5	9.29E+00	1.78E−01	6.88E+03	1.43E+02
11	82.5	8.79E+00	2.23E−01	9.79E+03	2.91E+02
12	87.5	6.89E+00	2.86E−01	1.36E+04	7.42E+02
13	92.5	4.35E+00	3.94E−01	1.94E+04	2.75E+03
14	97.5	2.32E+00	6.19E−01	4.00E+04	2.87E+04

a) Estimates are given in units of number of cancer cases per 100,000 person-years.

b) Middle points of the age intervals, 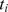, are given in years.

**Table 2:** Estimates of the population and individual hazard rates (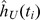
and 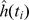, correspondingly) and their standard errors (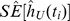 and 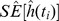) of PC occurrence in women.

Age intervals		Estimates of the hazard rates and their standard errorsa)			
Index	Middle pointb)	Population level		Individual level	
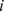	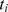	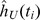	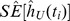	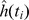	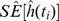
1	32.5	5.70E−02	6.52E−03	2.35E+01	2.69E+00
2	37.5	1.41E−01	1.06E−02	5.81E+01	4.37E+00
3	42.5	3.04E−01	1.63E−02	1.26E+02	6.75E+00
4	47.5	7.20E−01	2.67E−02	3.02E+02	1.12E+01
5	52.5	1.36E+00	3.90E−02	5.85E+02	1.67E+01
6	57.5	2.44E+00	5.57E−02	1.09E+03	2.50E+01
7	62.5	3.58E+00	7.21E−02	1.72E+03	3.47E+01
8	67.5	5.45E+00	9.36E−02	2.93E+03	5.12E+01
9	72.5	6.89E+00	1.13E−01	4.44E+03	7.55E+01
10	77.5	7.71E+00	1.32E−01	6.50E+03	1.20E+02
11	82.5	7.20E+00	1.50E−01	8.84E+03	2.11E+02
12	87.5	5.98E+00	1.77E−01	1.23E+04	4.61E+02
13	92.5	4.17E+00	2.26E−01	1.80E+04	1.47E+03
14	97.5	2.54E+00	3.37E−01	4.00E+04	1.43E+04

a) Estimates are given in units of number of cancer cases per 100,000 person-years.

b) Middle points of the age intervals, 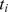, are given in years.

**Table 3:** Estimates of the population and individual hazard rates (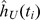
and 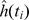, correspondingly) and their standard errors (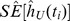 and 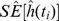) of PC occurrence in whites.

Age intervals		Estimates of the hazard rates and their standard errorsa)			
Index	Middle pointb)	Population level		Individual level	
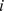	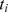	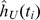	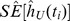	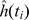	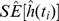
1	32.5	6.22E−02	5.14E−03	2.37E+01	1.96E+00
2	37.5	1.62E−01	8.55E−03	6.17E+01	3.26E+00
3	42.5	3.88E−01	1.39E−02	1.49E+02	5.32E+00
4	47.5	8.60E−01	2.20E−02	3.34E+02	8.53E+00
5	52.5	1.67E+00	3.25E−02	6.67E+02	1.29E+01
6	57.5	2.86E+00	4.54E−02	1.19E+03	1.90E+01
7	62.5	4.28E+00	5.97E−02	1.93E+03	2.71E+01
8	67.5	6.13E+00	7.66E−02	3.13E+03	3.98E+01
9	72.5	7.56E+00	9.29E−02	4.67E+03	5.98E+01
10	77.5	8.21E+00	1.09E−01	6.71E+03	9.69E+01
11	82.5	7.60E+00	1.28E−01	9.17E+03	1.78E+02
12	87.5	6.14E+00	1.54E−01	1.27E+04	4.04E+02
13	92.5	4.17E+00	2.01E−01	1.83E+04	1.35E+03
14	97.5	2.46E+00	3.05E−01	4.00E+04	1.33E+04

a) Estimates are given in units of number of cancer cases per 100,000 person-years.

b) Middle points of the age intervals, 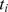, are given in years.

**Table 4:** Estimates of the population and individual hazard rates (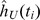
and 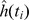, correspondingly) and their standard errors (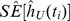 and 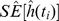) of PC occurrence in blacks.

Age intervals		Estimates of the hazard rates and their standard errorsa)			
Index	Middle pointb)	Population level		Individual level	
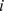	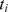	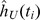	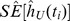	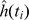	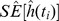
1	32.5	7.31E−02	1.46E−02	1.97E+01	3.94E+00
2	37.5	2.83E−01	3.06E−02	7.65E+01	8.26E+00
3	42.5	6.04E−01	4.81E−02	1.64E+02	1.31E+01
4	47.5	1.46E+00	8.24E−02	4.01E+02	2.27E+01
5	52.5	2.85E+00	1.27E−01	8.08E+02	3.62E+01
6	57.5	4.79E+00	1.84E−01	1.44E+03	5.55E+01
7	62.5	6.88E+00	2.44E−01	2.26E+03	8.10E+01
8	67.5	9.00E+00	3.04E−01	3.41E+03	1.18E+02
9	72.5	1.06E+01	3.82E−01	4.92E+03	1.86E+02
10	77.5	1.02E+01	4.41E−01	6.27E+03	2.90E+02
11	82.5	1.05E+01	5.86E−01	9.45E+03	6.12E+02
12	87.5	8.04E+00	7.16E−01	1.24E+04	1.40E+03
13	92.5	4.75E+00	8.58E−01	1.44E+04	3.51E+03
14	97.5	4.21E+00	1.44E+00	4.00E+04	3.70E+04

a) Estimates are given in units of number of cancer cases per 100,000 person-years.

b) Middle points of the age intervals, 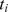, are given in years.

**Table 5:** Estimates of the population and individual hazard rates (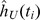
and 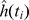, correspondingly) and their standard errors (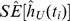 and 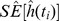) of PC occurrence in the Eastern geographic area.

Age intervals		Estimates of the hazard rates and their standard errorsa)			
Index	Middle pointb)	Population level		Individual level	
	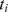	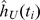	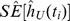	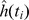	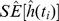
1	32.5	6.52E−02	6.67E−03	2.34E+01	2.39E+00
2	37.5	2.07E−01	1.22E−02	7.44E+01	4.40E+00
3	42.5	4.29E−01	1.84E−02	1.55E+02	6.66E+00
4	47.5	9.61E−01	2.93E−02	3.52E+02	1.07E+01
5	52.5	1.88E+00	4.34E−02	7.07E+02	1.63E+01
6	57.5	3.18E+00	6.03E−02	1.26E+03	2.39E+01
7	62.5	4.66E+00	7.84E−02	1.99E+03	3.38E+01
8	67.5	6.54E+00	9.98E−02	3.18E+03	4.94E+01
9	72.5	8.11E+00	1.22E−01	4.79E+03	7.50E+01
10	77.5	8.71E+00	1.42E−01	6.85E+03	1.22E+02
11	82.5	8.18E+00	1.68E−01	9.63E+03	2.31E+02
12	87.5	6.36E+00	1.97E−01	1.31E+04	5.23E+02
13	92.5	4.31E+00	2.56E−01	1.97E+04	1.85E+03
14	97.5	2.22E+00	3.59E−01	4.00E+04	1.74E+04

a) Estimates are given in units of number of cancer cases per 100,000 person-years.

b) Middle points of the age intervals, 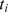, are given in years.

**Table 6:** Estimates of the population and individual hazard rates (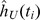
and 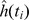, correspondingly) and their standard errors (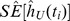 and 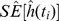) of PC occurrence in the Western geographic area.

Age intervals		Estimates of the hazard rates and their standard errorsa)			
Index	Middle pointb)	Population level		Individual level	
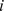	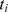	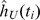	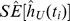	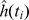	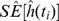
1	32.5	6.41E−02	7.22E−03	2.52E+01	2.84E+00
2	37.5	1.39E−01	1.11E−02	5.49E+01	4.38E+00
3	42.5	3.72E−01	1.92E−02	1.47E+02	7.59E+00
4	47.5	8.70E−01	3.15E−02	3.49E+02	1.26E+01
5	52.5	1.68E+00	4.68E−02	6.90E+02	1.93E+01
6	57.5	2.85E+00	6.61E−02	1.23E+03	2.87E+01
7	62.5	4.28E+00	8.77E−02	2.00E+03	4.14E+01
8	67.5	6.11E+00	1.12E−01	3.26E+03	6.11E+01
9	72.5	7.29E+00	1.35E−01	4.73E+03	9.13E+01
10	77.5	7.74E+00	1.58E−01	6.63E+03	1.47E+02
11	82.5	7.17E+00	1.87E−01	9.03E+03	2.70E+02
12	87.5	6.13E+00	2.36E−01	1.33E+04	6.65E+02
13	92.5	3.91E+00	2.99E−01	1.86E+04	2.19E+03
14	97.5	2.24E+00	4.47E−01	4.00E+04	2.15E+04

a) Estimates are given in units of number of cancer cases per 100,000 person-years.

b) Middle points of the age intervals, 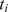, are given in years.

The comparison of the estimates 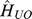 and 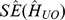 shows that men are more likely to get pancreatic cancer (PC) than women (i.e. the size of the pool of the individuals susceptible to PC, is bigger in men (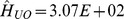; 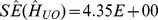) than in women (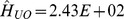; 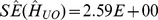). Analogously, blacks (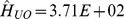; 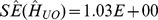) have a higher chance of getting PC than whites (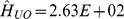; 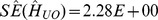). Finally, people living in the Eastern area (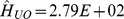; 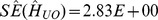) are more likely to get PC compared to those who live in the Western area (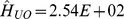; 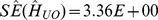). Comparison of the corresponding data indicates that all of these differences are statistically significant.

Values of 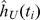 presented in Tables 1–6 suggest that, for the stratified populations, the estimates of the population hazard rates in all age intervals are nearly proportional: within the error limits, ratios of the corresponding 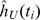 are nearly the same in all age intervals, except for a few points. The values of these ratios are close to the ratios of the corresponding 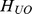. It should be noted that for the stratified populations with the same theoretical hazard function, 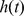, the proportionality of their 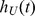 follows from formula (12). Since formula (12) was obtained assuming that the considered population has a dichotomous susceptibility cancer and because the prediction made by this formula is supported by the observed data, one can conclude that this assumption should be valid.

The empirical estimates of the individual hazard rates, 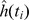, and their 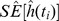 were obtained *via* 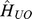, 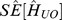, 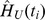 and 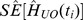 as described in Step 2 of the proposed computing framework (see Materials and Methods). The obtained estimates are given in Tables 1–6. It should be noted that the 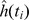 presented in these tables are also the estimates of the corresponding theoretical hazard functions of PC occurrence in the age intervals 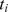 (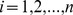). As can be seen from these tables, within the error limits, values of the 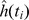, determined for the considered stratified populations are nearly the same and increase with age. Moreover, the obtained values of 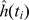
are very close to those that were determined for the occurrence of PC in the unstratified population (see Table 5 in [17]). Taken together, these data suggest the possibility of choosing “up” the same theoretical hazard function of PC occurrence for the stratified and unstratified populations. The choosing “up” the theoretical hazard function of cancer is required by Step 3 of the proposed computing framework (see Materials and Methods). In this work, for modeling the individual hazard function, 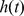, a three-parametric Weibull function, presented in Materials and Methods by formula (26), was utilized. A rationale for this choice is that this function was successfully used in [17] for PC modeling in the unstratified population.

Finally, values of the individual and population hazard rates of PC occurrence in the stratified populations were predicted using formulas (27) and (28), correspondingly. As the parameters of these functions, the following values were used: 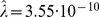, 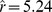 and 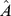 = 17. These values were determined for the PC occurrence in the unstratified population as suggested in Step 4 of the proposed computing framework. Note, such parametrical values were also obtained in [17] for the PC occurrence in the unstratified population. In other words, (independently on gender, race and geographic area of living) the pancreatic cancer can occur when an average number of clones developed from the mutated cells during the first year after the beginning of the effective period of the cancer exposure will be about 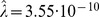 and a number of mutations transforming a normal cell into a malignant one will be about 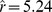. This cancer is clinically detected with a time shift (that includes a period between birth and the age at the beginning of carcinogenesis, as well as an average time needed for clonal expansion of malignant cells into the clinically detectable tumor [14]) of about 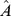 = 17 years.

**Figure 2:**
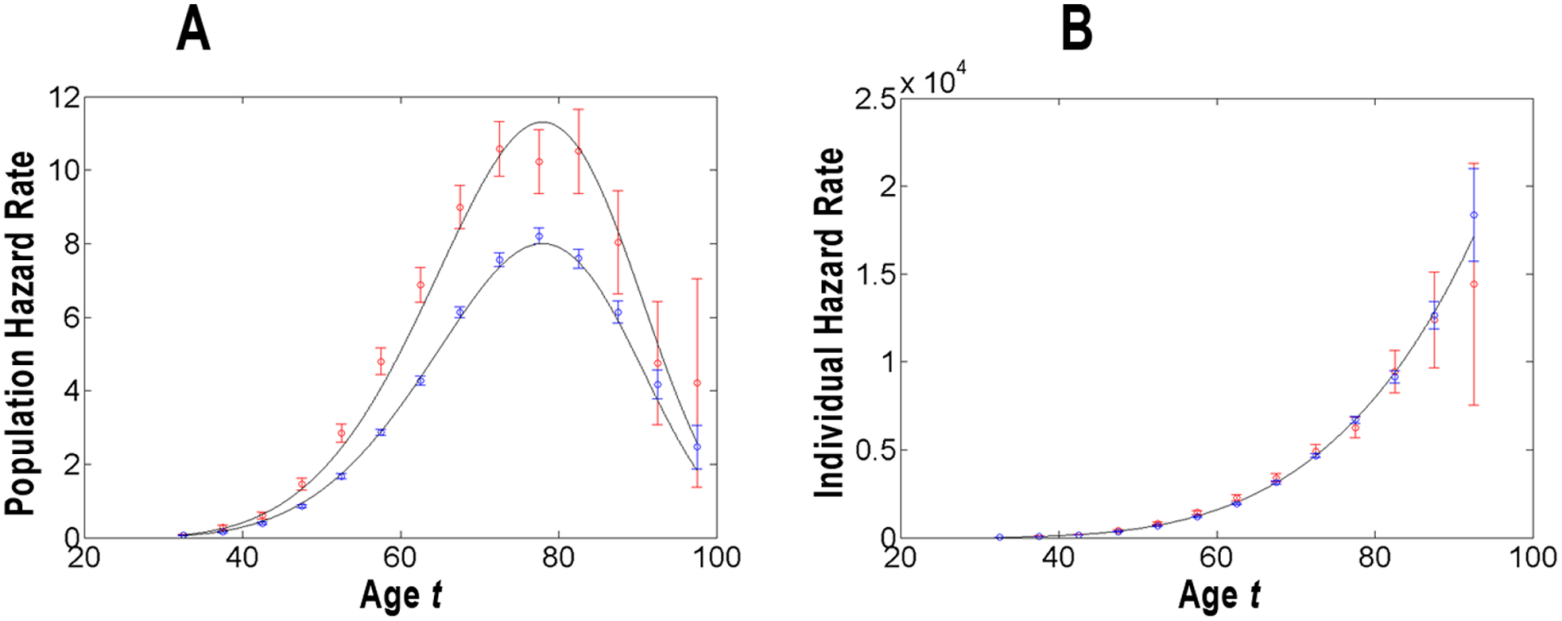
Modeling of the population (panel A) and individual (panel B) hazard rates of pancreatic cancer in whites and blacks. For these populations, the estimates of the rates and their 95% the confidence interval (*CI*) are given in units of number of cancer cases per 100,000 person-years and presented by circles and error bars of blue (for whites) and red (for blacks) vs. age in years. The modeled population and individual hazard functions are presented by solid lines.

**Figure 3:**
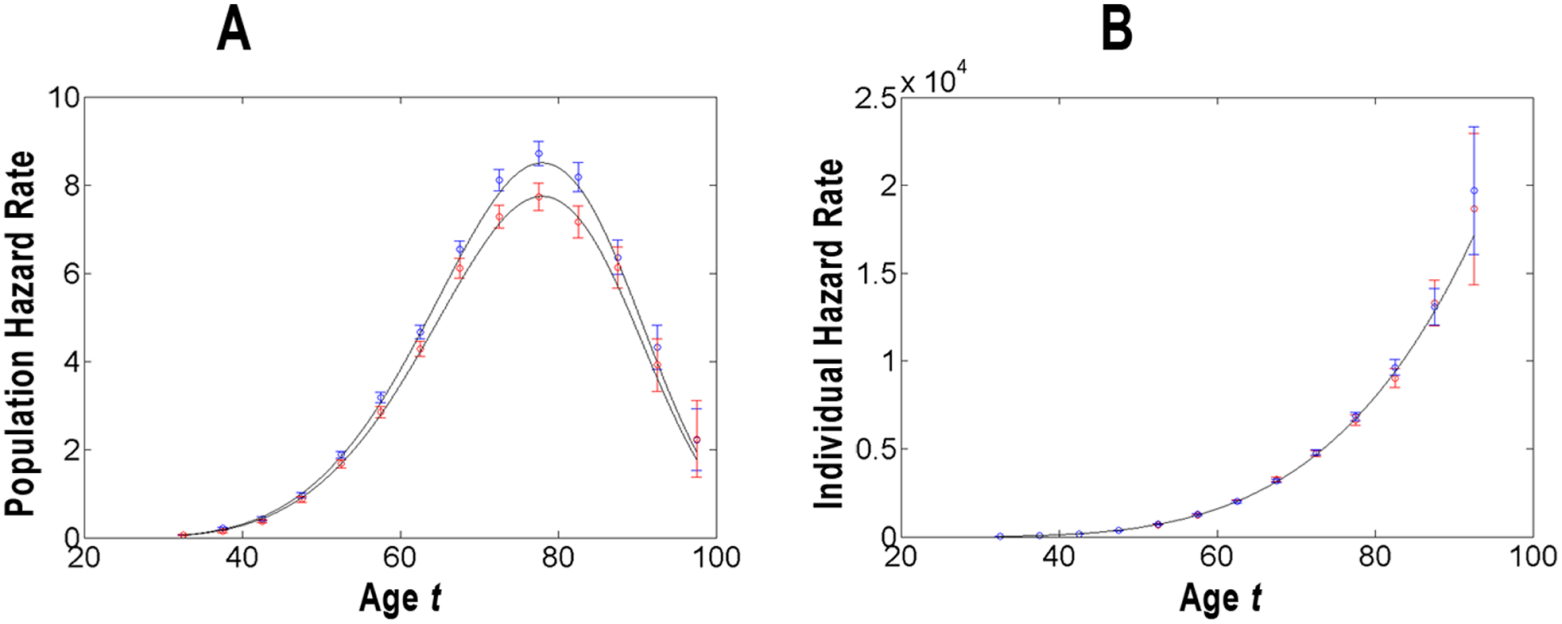
Modeling of the population (panel A) and individual (panel B) hazard rates of pancreatic cancer in the Eastern and Western geographic areas. For these populations, the estimates of the rates and their 95% the confidence interval (*CI*) are given in units of number of cancer cases per 100,000 person-years and presented by circles and error bars of blue (for Eastern area) and red (for Western area) vs. age in years. The modeled population and individual hazard functions are presented by solid lines.

Figures 1–3 graphically present the estimates of the population (panel A) and individual (panel B) hazard rates, as well as the modeled population and individual hazard functions of PC occurrence in the populations stratified by gender, race and geographic era of living. The estimates of the rates are shown for middle points of the corresponding age intervals 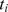 and are given in units of number of cancer cases per 100,000 person-years. The error bars indicate 95% of the confidence interval (*CI*). In panels B, the points at 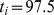 are omitted because of the large error bars. In the Figures 1–3, the modeled values of the population and individual hazard functions are shown by solid lines. The modeled individual and population hazard functions are presented by solid lines.

Visual inspection of Figures 1–3 suggests that the predicted curves, 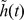
and 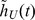, (with a first-order approximation) well approximate the corresponding observed data, 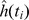
and 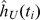, correspondingly. Thus, one can conclude that the PC model, developed for the unstratified population with a dichotomous susceptibility to cancer, well predicts the values of the population hazard rate of PC for the populations stratified by gender, race, and the geographical area of living.

## Conclusions

In this work, a novel, “top-down” computing approach for carcinogenic modeling is developed. This approach is based on a general assumption that, in the population, only a small fraction of individuals susceptible to cancer will eventually get cancer in their lifetime. It allows for decomposing of the mathematical problem of the carcinogenic modeling on two more simple problems. The first (inverse) problem is to determine the values of the age-specific hazard rate in individuals susceptible to cancer (individual hazard rate) by the age-specific hazard rate observed in the population (population hazard rate). The second (direct) problem is to predict the age-specific hazard rate in individuals susceptible to cancer by a chosen “up” theoretical hazard function. The three-parametric Weibull function is utilized.

The proposed approach was applied for carcinogenic modeling of pancreatic cancer (PC) in populations stratified by gender, race and geographic area of living. The performed modeling suggested that, in the stratified populations, the population hazard rate of PC has turnover at the age of ∼77 years and then the population hazard rate falls at older ages, while the individual hazard rate of PC are continuously increasing in age. In the frame of the proposed model, this phenomenon is explained by the fact that the pool of individuals susceptible to PC progressively diminishes with age. The size of the pool of individuals susceptible to PC (i.e. the probability to get PC) is bigger for men *vs*. women, for blacks *vs*. whites, and for those who live in the Eastern *vs*. Western geographic areas. The sizes of the pools of individuals susceptible to PC proportionally influence the population hazard rate, but do not influence the individual hazard rate of PC. For the unstratified population and for the populations stratified by the considered categorical variables, the estimates of the individual hazard rates of PC were nearly the same, suggesting a possibility of using the same three-parametric Weibull function for their approximation. The values of the parameters of this function, obtained for the unstratified population, were used to predict the values of the population hazard rate for the stratified populations. To make these predictions, a size of the corresponding pool of individuals susceptible to cancer estimated from the observed data was used. The observed population hazard rates were well approximated by the corresponding predicted population hazard functions. This suggests that the PC model for the unstratified population can be used for the populations stratified by gender, race and the geographic area of living while predicting their individual and population hazard rates.

Overall, this work demonstrates that an acceptance of the hypothesis of the dichotomous susceptibility to cancer in the population radically changes and enhances the computing framework currently used in carcinogenic modeling. Moreover, this hypothesis implies that a mechanism that regulates dichotomous susceptibility to cancer (rather than the commonly believed aging and/or mutation mechanisms) should be considered as a main driving force of carcinogenesis.
